# Impact of temperature on vector competence of *Culex pipiens molestus*: implications for Usutu virus transmission in temperate regions

**DOI:** 10.1186/s13071-025-06948-z

**Published:** 2025-07-29

**Authors:** Nicola Seechurn, Jack Pilgrim, Ken Sherlock, Jolanta Tanianis-Hughes, Marcus Blagrove, Grant L. Hughes, Jolyon M. Medlock, Nicholas Johnson, Matthew Baylis

**Affiliations:** 1 Department of Livestock and One Health, Institute of Infection, Veterinary and Ecological Sciences, University of Liverpool, Liverpool, UK; 2 Department of Vector Biology and Tropical Disease Biology, Centre for Neglected Tropical Diseases, Liverpool School of Tropical Medicine, Liverpool, UK; 3 UK Field Epidemiology Training Programme Field Service, South East and London, UK Health Security Agency, London, UK; 4 Vector Borne Diseases, Virology Department, Animal and Plant Health Agency (APHA), Woodham Lane, Surrey, KT15 3NB UK

**Keywords:** *Culex pipiens molestus*, Vector competency, Usutu virus

## Abstract

**Background:**

Usutu virus (USUV) has been detected annually in the southeast of England since 2020. USUV RNA has been identified in wild birds and mosquito populations, and exposure of captive birds to USUV has also been confirmed in the UK. Since its first detection in London, USUV’s distribution has expanded across the South East, highlighting necessity to understand USUV transmission dynamics in the UK. The primary vectors of USUV in the UK are likely *Culex pipiens* mosquitoes. *Culex pipiens molestus* is one biotype which shows no restriction in host preference and may play an important role in transmitting USUV from birds to humans.

**Methods:**

A laboratory colony of *Cx. pipiens molestus* mosquitoes were orally infected with the London strain of USUV and incubated at 22 ℃, 20 ℃ and 18 ℃ for up to 28 days. Body samples and mosquito saliva samples were collected and analysed using a quantitative real-time reverse transcription PCR to determine infection and transmission potential, respectively.

**Results:**

USUV RNA was detected in all sample times at all temperatures assessed, with the 22 ℃ showing the greatest proportion of saliva and body positive samples. At this temperature, there was also an eight-fold increase in the relative viral copy number in the mosquito bodies, which was unobserved at other experimental temperatures. When a more sensitive PCR assay was used at the lowest experimental temperature used (18 ℃), USUV RNA was present in the mosquito saliva and body samples for longer and showed a greater proportion of positive samples compared to 20 ℃.

**Conclusions:**

This study has demonstrated that *Cx. pipiens molestus* may be able to transmit USUV at 22 ℃. Active replication of USUV was identified in the mosquito bodies at 22 ℃ but not at lower temperatures, suggesting that 20 ℃ to 22 ℃ may be an important threshold in USUV replication and transmission. Utilisation of a more sensitive assay for the lower experimental temperatures revealed that USUV was detectable at 18 ℃. Therefore, when conducting infection studies on temperate mosquito-borne viruses, it is important to consider assay sensitivity.

**Graphical Abstract:**

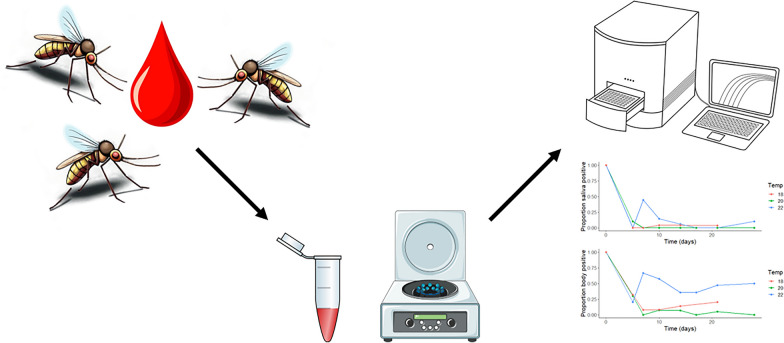

**Supplementary Information:**

The online version contains supplementary material available at 10.1186/s13071-025-06948-z.

## Background

In the summer of 2020, USUV was first identified in five Eurasian blackbirds (*Turdus merula*) and one house sparrow (*Passer domesticus*) in the southeast of England. Identification of USUV in UK non-migratory bird species, such as house sparrows and Eurasian blackbirds, suggests autochthonous transmission by UK-endemic mosquitoes [1]. Further surveillance in this area in 2021 identified the presence of USUV in another Eurasian blackbird, suggesting overwintering of USUV and possible further autochthonous transmission [2]. USUV was later detected in rural Cambridgeshire in 2023, demonstrating expansion of the USUV distribution since its first identification [3]. USUV has become endemic in northern and central Europe; however, in these countries, summer daily temperatures tend to be higher than in the UK. Repeated detection of USUV over multiple years suggests that UK summer temperatures are permissive. The aim of this work is to determine a threshold at which replication and potential transmission can occur by UK native mosquitoes.

*Culex pipiens* s.l. is the main European USUV vector and is a species complex comprised of *Culex pipiens pipiens*, *Culex pipiens molestus* and hybrid forms. *Culex pipiens pipiens* is widely distributed and highly abundant across the UK and is the predominant enzootic vector of USUV through circulation of virus between avian and *Culex* mosquito populations [4]. By contrast, *Cx. pipiens molestus* is a voracious human-biting biotype with a distribution mostly limited to underground locations, including the London Underground [5]. This biotype is a potential bridge vector of USUV, being able to spread the virus from birds to humans. Where *Cx. pipiens pipiens* and *Cx. pipiens molestus* have occurred sympatrically in surface habitats, hybridisation can occur [6, 7]. These hybrid species have been described as opportunistic feeders and do not have strict host preferences, leading to their potential as additional bridge vectors [8].

Vector competency for USUV has been evidenced in multiple mosquito species: *Cx. torrentium*, *Cx. quinquefasciatus*, *Cx. restuans*, *Cx. neavei*, *Cx. pipiens molestus*, *Cx. pipiens* s.l., *Cx. pipiens pipiens*, *Aedes japonicus japonicus* and *Ae. albopictus* [9–13]. A previous infection study using a UK-derived laboratory colony of USUV demonstrated infection, dissemination and transmission in a single individual mosquito infected with the African reference strain of USUV, suggesting a strong infection barrier to UK *Culex* mosquitoes for this strain. Successful dissemination and transmission has been demonstrated in other vector competency studies under different experimental conditions [10–12]. Additionally, levels of vector competency have been shown to vary between the two *Culex pipiens* s.l. biotypes, *Cx. pipiens pipiens* and *Cx. pipiens molestus* [14].

Given the gap in knowledge of vector competence of USUV in cooler regions, we aimed to investigate the effect of vector competency of *Cx. pipiens molestus* under temperate conditions. According to climate data collected by the Met office, average July temperatures have reached 20 ℃ historically; therefore, temperatures above and below this figure were used as the incubation temperatures [15]. The first study (‘Study 1’) was conducted at 22 ℃ and 20 ℃ and aimed to assess the vector competency of a laboratory colony of *Cx. pipiens molestus* at these temperatures. The second study (‘Study 2’) aimed to determine whether the virus was detectable < 20 ℃ when assay sensitivity was increased. Overall, this infection study assesses the vector competency and importance of assay sensitivity in a laboratory colony of *Cx. pipiens molestus*, incubated between 18 ℃ and 22 ℃, using the USUV London strain of the African 3 lineage [1].

## Methods

### Mosquito rearing and virus strain

Viral stocks of USUV London were received from the Animal and Plant Health Agency (APHA) at a titre of 4 × 10^8^ PFU/ml. Virus stock was shipped on dry ice and was stored at – 80 ℃ upon arrival. *Culex pipiens molestus* mosquito eggs were received from The Pirbright Institute [16] and were reared. To generate sufficient mosquitoes of a similar age for each experiment, mosquitoes were exposed to the Haemotek feeder containing defibrinated horse blood (E & O Laboratories) overnight; the following F1 generations were used for infection studies. The mosquito colony was maintained in the Insectary Facilities at Leahurst Campus, University of Liverpool.

### Oral infection of *Cx. pipiens molestus* with USUV

Mosquitoes were orally infected with a USUV titre of 4 × 10^7^ PFU/ml. A Haemotek feeder containing USUV and defibrinated horse blood was placed in the BugDorm cage for 3 h. Mosquitoes were fed in the dark, and non-bloodfed mosquitoes were removed. One hundred and thirty-eight *Cx. pipiens molestus* mosquitoes were infected with USUV at a titre of 4 × 10^7^ PFU/ml and incubated at 20 ℃ and 18 ℃ and 118 mosquitoes were infected and incubated at 22 ℃. Following infection with USUV, mosquitoes were placed into DispoSafe pots and labelled with the number of days post infection on which they would be sampled.

### Time series and incubation temperatures

*Culex pipiens molestus* mosquitoes were incubated at 18 °C, 20 °C and 22 °C using Sanyo TM MIR-154 incubators. Incubators were assessed prior to each experiment to check that the appropriate temperature could be maintained by the incubator. Incubation temperatures were monitored throughout experiments using a thermometer permanently placed in the incubator. Samples were collected immediately after feeding (0 days post-infection [dpi]) and 5, 10, 14, 17, 21 and 28 dpi.

### Forced salivation and sample collection

FlyNap (Blades Biological Limited, Kent) was used to anaesthetise the mosquitoes for forced saliva extraction. Once anaesthetised, mosquito proboscises were placed into a capillary tube containing mineral oil for 30 min. After 30 min, the mosquito bodies were placed into an Eppendorf tube containing 250 μl of Trizol (Thermo Fisher Scientific). The mosquito saliva was removed from the capillary tube using a micropipette and placed into a separate Eppendorf tube containing 100 μl of Trizol (Thermo Fisher Scientific).

### RNA extraction

Following placement of bodies and saliva into Trizol, RNA was extracted as per the manufacturer’s instructions. The RNA pellets was resuspended in 20 µl of nuclease-free water for 22 ℃ and 20 ℃ and 10 µl for 18 ℃.

### Real-time reverse transcription PCR (RT-PCR)

Real-time RT-PCR was undertaken using a Roche 480 LightCycler (Roche, Basel, Switzerland). The total volume per well was 10 μl. This was made up of 5 μl of Master Mix, 0.4 μl of 10 μM forward (CGTGAAGGTTACAAAGTCCAGA) and reverse primers (TCTTATGGAGGGTCCTCTCTTC) targeting the nonstructural protein 1 gene, 0.2 μl of 10 μM probe, 1.9 μl of nuclease-free water, 0.5 μl of RT-mix and 2 μl of RNA template. RNA extract from a male *Cx. pipiens molestus* was used as a no-template control (NTC) and nuclease-free water was used as a negative control (NC). A reverse transcription step was undertaken at 50 ℃ for 30 min, followed by an initial activation step at 95 ℃ for 15 min. This was followed by 45 cycles of denaturation and combined annealing/extension at 94 ℃ for 15 s and 60 ℃ for 1 min. USUV nucleic acid, SAAR 1776 strain (BEI resources, Bethesda, MD, USA), was used as a positive control (PC). Saliva samples were run in triplicate and whole-body samples in duplicate on a 96-well real-time PCR plate. Half of the elution volume was used for 18 ℃ compared to 20 ℃ and 22 ℃ to increase the sensitivity of this assay.

### Standard curve

Standards were produced by Integrated DNA Technologies using the NS1 sequence obtained from GenBank. Standards were serially diluted 1 in 10 from 1.7 × 10^12^ copies/μl to 1.7 × 10^2^ copies/μl, with a reaction efficiency of 1.832. Viral copy number was calculated using the standard curve for all samples with a crossing point value < 40. Data for standard curve generation and minimum detection limit are as described by Seechurn [17].

### Data analysis

Data were analysed using Rstudio [18]. Figures were produced using the ggplot and tidyverse packages [19, 20]. Survival analysis was undertaken using the ggsurvfit and survival packages [20, 21].

## Results

### Mortality rates and survival analysis for 22 ℃, 20 ℃ and 18 ℃

**Fig. 1:**
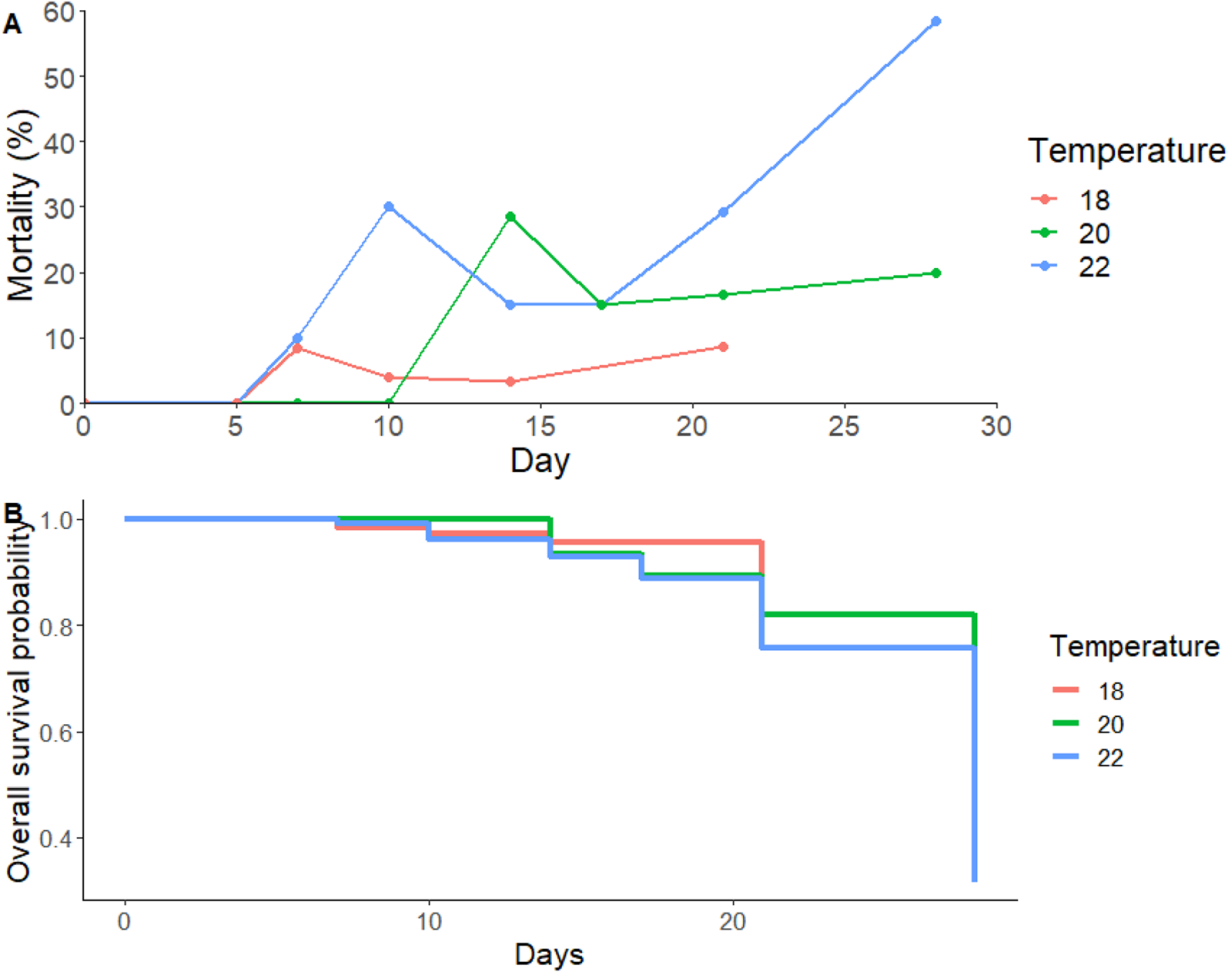
Total percentage mortality of *Culex pipiens molestus* at day of sampling (0, 5, 7, 10, 14, 21 and 28 days post infection). Mosquitoes were incubated up to 28 days for 22 ℃ (*n* = 118) and 20 ℃ (*n* = 138) and up to 21 days for 18 ℃ (*n* = 138). Mosquito samples were collected at 0 dpi (*n* = 5 [22 ℃]; *n* = 9 [20 ℃]; *n* = 14 [18 ℃]), 5 dpi (*n* = 5 [22 ℃]; *n* = 10 [20 ℃]; *n* = 10 [18 ℃]), 7 dpi (*n* = 10 [22 ℃]; *n* = 15 [20 ℃]; *n* = 24 [18 ℃]), 10 dpi (*n* = 10 [22 ℃]; *n* = 14 [20 ℃]; *n* = 25 [18 ℃]), 14 dpi (*n* = 20 [22 ℃]; *n* = 21 [20 ℃]; *n* = 30 [18 ℃]), 17 dpi (*n* = 20 [22 ℃]; *n* = 20 [20 ℃]), 21 dpi (*n* = 24 [22 ℃]; *n* = 24 [20 ℃]; *n* = 35 [18 ℃]) and 28 dpi (*n* = 24 [22 ℃]; *n* = 25 [20 ℃]). Percentage mortality was reported per DispoSafe pot in which infected mosquitoes were incubated (**A**). Kaplan-Meier plot demonstrating overall survival probability throughout experimental temperatures used. No left or right censoring occurred as all mosquitoes were used in analysis. Mosquitoes were incubated for up to 28 days for 22 ℃ and 20 ℃ and for 21 days for 18 ℃ (**B**)

Three trends were observable in mortality rates identified in this experiment (Fig. 1A); first, there was an increase in mortality over time for all temperatures conducted; second, an early peak in mortality followed by decline and then further increase was observed at all temperatures; third, higher levels of mortality were observed at higher temperatures.

Using the same mortality data, the probability of survival at different time points was analysed with a Kaplan-Meier plot (Fig. 1B). Survival analysis using the Cox proportional hazards model indicated that temperature had an effect on overall chance of survival, with statistical significance identified between 20 ℃ and 22 ℃ (*p* < 0.05).

### Study 1 (Temperatures: 22 ℃ and 20 ℃)

#### Proportion body and saliva positive

USUV RNA was detected in both bodies and saliva at 22 ℃ and 20 ℃ (Table 1). Data in graphical form can be found in Additional file 1: Figure A1. At both temperatures, 100% of mosquito bodies were positive at 0 dpi. Thereafter, the proportion positive dropped substantially before starting to rise again. The rise started earlier and reached a higher level at 22 ℃ compared to 20 ℃. The time taken to reach the greatest proportion of body positive increased as the experimental temperature decreased, with 7 dpi, and 10 dpi being the time point for greatest proportion of infected bodies at 22 ℃ and 20 ℃, respectively.

**Table 1:** Summary of USUV RNA detection in mosquito and saliva of *Culex pipiens molestus* at 22 ℃ and 20 ℃

Temperature (℃)	Day	Sample type	Positive samples	Total sampled	Proportion positive
22	0	Saliva	0	5	0.00
22	0	Body	5	5	1.00
22	5	Saliva	0	5	0.00
22	5	Body	1	5	0.20
22	7	Saliva	4	9	0.44
22	7	Body	6	9	0.67
22	10	Saliva	1	7	0.14
22	10	Body	4	7	0.57
22	14	Saliva	1	17	0.06
22	14	Body	6	17	0.35
22	17	Saliva	0	16	0.00
22	17	Body	6	17	0.35
22	21	Saliva	0	17	0.00
22	21	Body	8	17	0.47
22	28	Saliva	1	10	0.10
22	28	Body	5	10	0.50
20	0	Saliva	0	9	0.00
20	0	Body	9	9	1.00
20	5	Saliva	1	10	0.10
20	5	Body	3	10	0.30
20	7	Saliva	0	15	0.00
20	7	Body	0	15	0.00
20	10	Saliva	0	14	0.00
20	10	Body	1	14	0.07
20	14	Saliva	0	15	0.00
20	14	Body	1	15	0.07
20	17	Saliva	0	17	0.00
20	17	Body	0	17	0.00
20	21	Saliva	0	20	0.00
20	21	Body	1	20	0.05
20	28	Saliva	0	19	0.00
20	28	Body	0	19	0.00

At both temperatures, 100% of mosquito saliva samples were positive at 0 dpi. At 22 ℃, the proportion of saliva positives dropped to near zero by 5 dpi and then increased to a maxima at 7 dpi, with slow decline after this, before a further increase at 28 dpi. At 20 ℃, the percentage positive declined to zero by 7 dpi with no further detection of USUV after 7 dpi.

#### Relative viral copy number in mosquito bodies

**Fig. 2:**
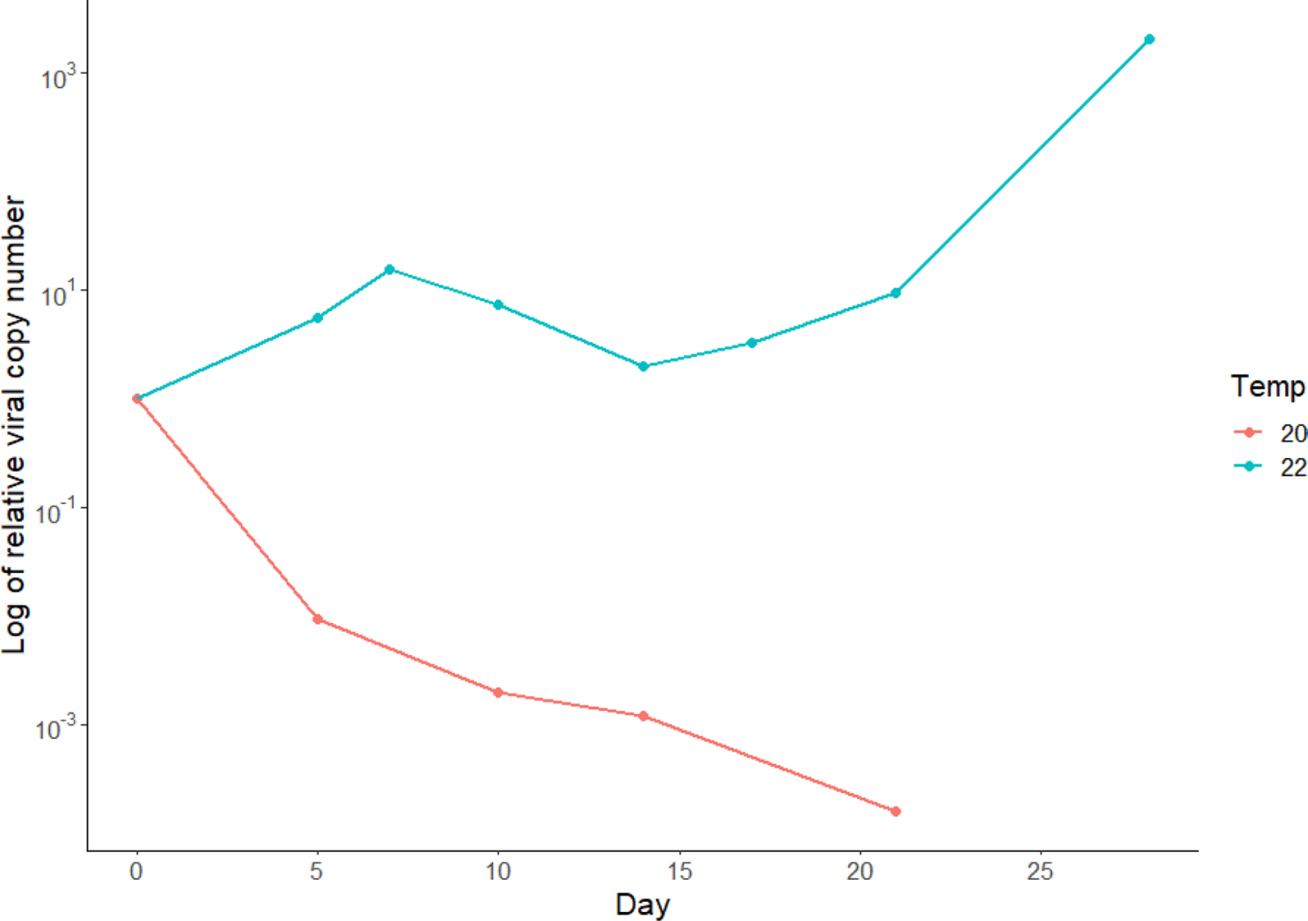
Change in viral copy number within mosquito bodies over time, relative to 0 dpi, at 22 ℃ and 20 ℃. Data provided on log scale. Each data point represents the mean titre of positive mosquitoes at each time point. Pools of mosquitoes were fed spiked blood containing USUV at a titre of 4 × 10^7^ PFU/ml and were tested by real-time RT-PCR. Positive mosquito samples were assessed for viral titre at 0 dpi (*n* = 5/5^a^ [22 ℃]; *n* = 9/9 [20 ℃]), 5 dpi (*n* = 1/5 [22 ℃]; *n* = 3/10 [20 ℃]), 7 dpi (*n* = 6/9 [22 ℃]; *n* = 0/15 [20 ℃]), 10 dpi (*n* = 4/7 [22 ℃]; *n* = 1/14 [ 20 ℃]), 14 dpi (*n* = 6/17 [22 ℃]; *n* = 1/15 [20 ℃]), 17 dpi (*n* = 6/17 [22 ℃]; *n* = 0/17 [20 ℃]), 21 dpi (*n* = 8/17 [22 ℃]; *n* = 1/20 [20 ℃]) and 28 dpi (*n* = 5/10 [22 ℃]; *n* = 0/19 [20 ℃]). ^a^The numerator represents positive mosquito sample out of total number of mosquitoes tested at each time point

The log of the viral copy number at each time point in mosquito bodies was compared to the log of the starting viral copy number (0 dpi) to determine how the viral copy number changed throughout the experiment. For the purpose of this study, the change in viral copy number over the experimental period is named the relative viral copy number. Viral copy number was calculated per mosquito. At 20 ℃, the log of the relative copy number decreased at each time point to be over three times smaller by 21 dpi. By contrast, the log of the relative copy number increased at 22 ℃, albeit with evidence of a decrease between 7 and 14 dpi. By 28 dpi, the log of the relative viral copy number was over three times higher than at the beginning of the experimental period (Fig. 2). Relative viral copy number in saliva samples was not assessed as low numbers of positive saliva samples were observed with low viral copy numbers of USUV.

### Study 2 (Temperature: 18 ℃)

#### Proportion body and saliva positive

At 18 ℃, and using a more sensitive detection method, all bodies were positive at 0 dpi. This then declined to a minimum at 7 dpi before increasing again to 21 dpi (Table 2). Data in graphical form can be found in Additional file 2: Figure A2. All saliva samples were positive at 0 dpi, but the percent positive was zero at 5 dpi. As expected, the proportion of saliva positive was consistently less than the proportion of body positive. Compared to 18 ℃, USUV RNA was only detectable in saliva samples at 5 dpi in mosquitoes held at 20 ℃ but was detectable at 10, 14 and 21 dpi at 18 ℃ using the more sensitive assay.

**Table 2:** Summary of USUV RNA detection in mosquito and saliva of *Culex pipiens molestus* at 18 ℃

Day	Sample type	Positive samples	Total sampled	Proportion positive
0	Saliva	0	15	0.00
0	Body	15	15	1.00
5	Saliva	0	19	0.00
5	Body	6	19	0.32
7	Saliva	1	14	0.05
7	Body	1	13	0.07
10	Saliva	1	24	0.00
10	Body	2	24	0.08
14	Saliva	1	26	0.04
14	Body	4	29	0.14
21	Saliva	1	30	0.03
21	Body	6	30	0.21

#### Relative viral copy number in mosquito bodies

**Fig. 3:**
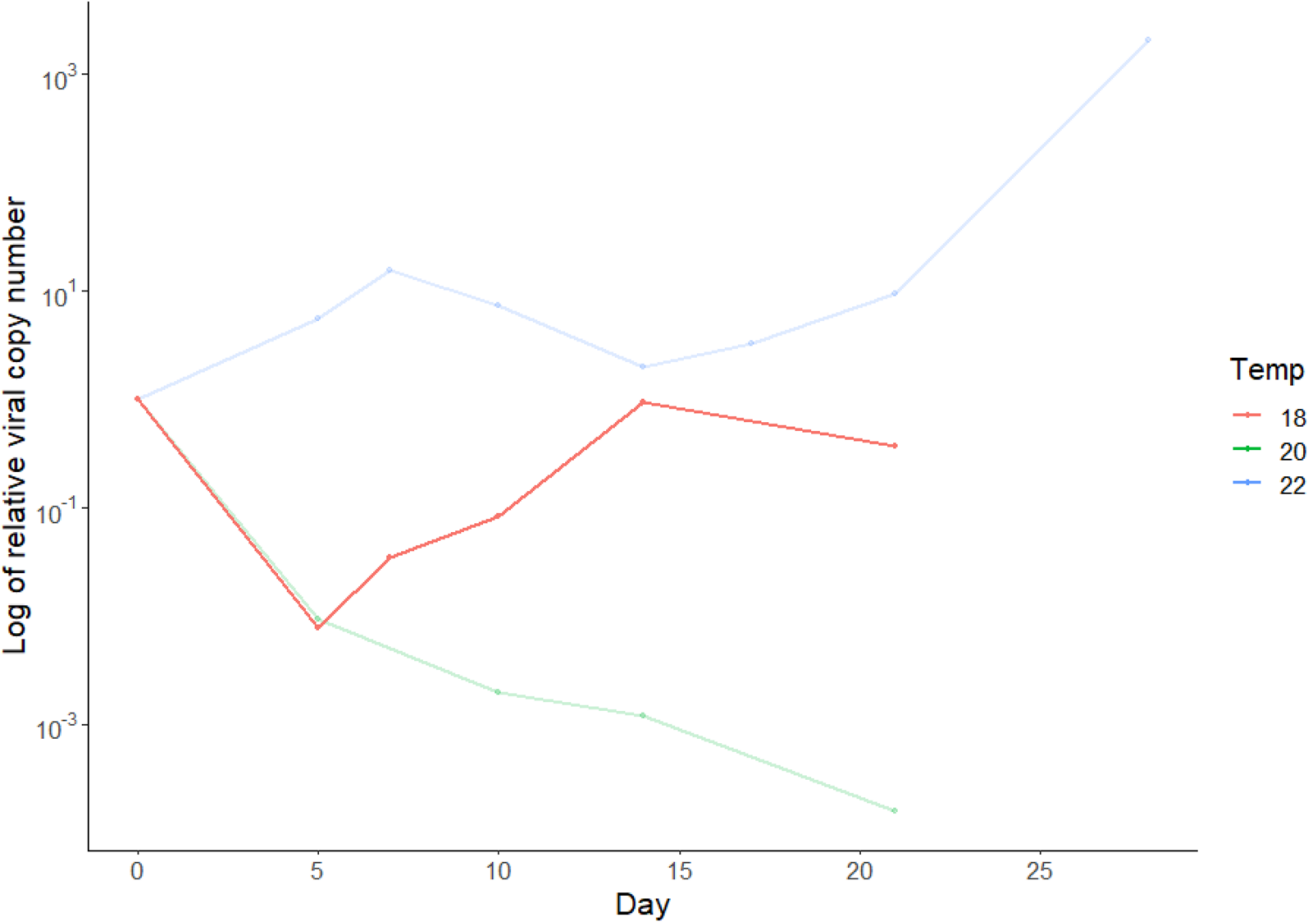
Demonstration of the log of the relative viral copy number within mosquito bodies over time, relative to day 0 viral copy number, at 22 ℃, 20 ℃ and 18 ℃. Data provided on log scale. Pools of mosquitoes were fed virus spiked blood containing USUV at a titre of 4 × 10^7^ PFU/ml and were tested by real-time RT-PCR. Each data point represents the mean titre of positive mosquitoes at each time point. Volume of elution buffer was halved for samples incubated at 18 ℃. Positive mosquito samples were assessed for viral titre at 0 dpi (*n* = 0/15^a^), 5 dpi (*n* = 6/19), 7 dpi (*n* = 1/13), 10 dpi (*n* = 2/24), 14 dpi (*n* = 4/29), 21 dpi (*n* = 6/30). ^a^The numerator represents the positive mosquito sample out of the total number of mosquitoes tested at each time point

The relative viral copy number at 18 ℃ initially decreased between 0 and 5 dpi and then increased back to starting relative viral copy number at 14 dpi. A small decrease in relative viral copy number was then observed at 21 dpi (Fig. 3). The experiment undertaken at 20 ℃, as previously described, demonstrated a continuous decline in relative viral copy number throughout all time points, with the lowest relative viral copy number occurring at 21 dpi. The fluctuation of relative viral copy number at 18 ℃ is more similar to 22 ℃, although relative viral copy number at 22 ℃ increased to much higher levels than at the start of the experimental period.

## Discussion

This study demonstrates the ability of a laboratory colony of *Cx. pipiens molestus* to become infected with, disseminate and be able to transmit the London strain of USUV under UK ambient temperatures. This has been achieved through detection of USUV RNA in mosquito bodies and saliva, with the greatest proportion of saliva positive samples occurring at 22 ℃. This study suggests that temperatures in the range of 20 ℃ to 22 ℃ provide an important threshold for USUV replication. Additionally, we have demonstrated that temperatures of 19 ℃ and 20 ℃ may facilitate transmission of the London strain of USUV [22]. The picture is more complex, however, as when a more sensitive detection method was used, the proportion of mosquitoes infected and the number of viral genome copies both increased even at the low temperature of 18 ℃. Lower viral copy numbers can be potentially transmitted in mosquito saliva at cooler temperatures, and incorporation of a more sensitive assay in these experiments may lead to a reduction in false-negative results. The strong effect of temperature on USUV infection supports that the vector competency and capacity of *Culex pipiens molestus* mosquitoes to transmit USUV may increase as temperatures rise. However, it is likely this relationship is not linear and that there may be a rise in extreme temperature which does not facilitate viral replication and may have detrimental effects on other elements required for successful pathogen transmission [23].

The data presented in this study produce contrasting results compared to another study which infected a laboratory colony of *Cx. pipiens* s.l. with the SAAR-1776 African strain of USUV. This study suggested a significant infection barrier of UK *Cx. pipiens* s.l. to this African strain. The difference in infection observed in these two studies may be suggestive of an evolutionary adaptation of USUV London strain to be transmitted by *Cx. pipiens* s.l. mosquitoes in temperate regions. The evidence presented here suggests that successful viral replication can occur in mosquito bodies at 22 ℃. USUV was detected in mosquito saliva at temperatures as low as 18 ℃ but viral titre in mosquito bodies at 20 ℃ and 18 ℃ did not exceed the viral titre at day 0, suggesting proliferation of USUV could not occur at temperatures < 20 ℃. Given higher relative viral copy numbers were identified at 18 ℃ compared to 20 ℃ when a more sensitive assay was used, this highlights the importance of using sensitive assays for future studies focusing on USUV transmission in temperate regions.

This study has identified evidence of an eclipse phase in mosquito bodies. This is evidenced with a decrease in proportion positive and decrease in viral titre, followed by an increase in both values at 22 ℃ and at 18 ℃. This demonstrates the subsequent decline of virus, as the virus in the blood meal is digested or enters midgut cells but is not yet replicating in the haemocoel or salivary glands. At all temperatures, 100% of mosquito body samples were positive at 0 dpi; this is likely from residual virus in the mouthparts from the ingested blood meal. Relative viral copy number in saliva samples was not assessed as low numbers of positive saliva samples were generated suggesting very low viral copy numbers of USUV in saliva samples. Relatively small sample sizes (5 to 30 mosquitoes per time point) were sampled at each time point in the various temperatures assessed. However, the number of infected mosquitoes sampled was nevertheless higher than in other infection studies with USUV [24].

The experimental model used here did not allow for fluctuations in temperature, as would occur naturally during the day and night. These temperature changes may have large consequences for viral replication and should be considered in future experiments. Effect of fluctuating temperatures on vector competency has been investigated regarding West Nile virus (WNV) and *Cx. quinquefasciatus* and *Culex tarsalis* mosquitoes [25]. McGregor et al. [25] showed that daily fluctuating temperature had a significant effect on viral titers in these mosquito species. Additionally, daily fluctuating temperatures have been shown to effect development time, fecundity and adult lifespan in *Culex pipiens molestus* [26]. Further work may also assess how behavioural effects of infected mosquitoes can impact on viral replication given that mosquitoes will identify desirable microclimates.

A real-time RT-PCR assay was used to detect USUV in mosquitoes but, as this assay detects fragments of genome, the viability of virus within samples tested could not be determined. The presence of viable virus would normally be demonstrated by plaque assay (with live virus killing cells, if present) but that was not undertaken here. Hence, despite finding positive USUV saliva by RT-PCR, it is not proven that there was the possibility of transmission of USUV at any temperature. Plaque assays were not undertaken because collected samples were placed immediately into Trizol, which kills live viruses. Nevertheless, there is a strong positive correlation of viral quantification through real-time RT-PCR and plaque assays for yellow fever virus (YFV), a flavivirus related to USUV, demonstrating some inference of infectivity may be possible when using real-time RT-PCR [27].

## Conclusions

This study has demonstrated the vector competency of a laboratory colony of *Cx. pipiens molestus* for the London strain of USUV, an African lineage 3 strain. Here, we demonstrate a critical temperature threshold between 22 ℃ and 20 ℃, demonstrating active USUV replication and potential transmission at 22 ℃. We highlight the possibility of permissiveness of transmission of USUV in London and, more broadly, the southeast of England. We highlight the importance of assay sensitivity when performing vector competency studies at cooler temperatures. Furthermore, preliminary data has been provided in this study which can be used to inform future infection studies which have the aim of extracting extrinsic incubation period (EIP) information to inform epidemiological and disease-risk models. We suggest that sustained transmission is possible at current ambient temperatures, and considering climate change, it is possible the areas of risk of USUV transmission in the UK may expand in coming years.

## Supplementary Information

Additional file 1. Figure A1: Proportion of body and saliva positive samples at 22 ˚C and 20 ˚C. Saliva samples at 0 dpi were positive, likely from virus contamination in the mouthparts, and so here are forced to one (A). Body samples at 0 dpi were positive, likely from virus in the bloodmeal (B). Pools of mosquitoes were fed spiked blood containing USUV at a titre of 4 × 10^7^ PFU/ml and were tested by real-time RT-PCR.

Additional file 2. Figure A2: Proportion of body positive and saliva positive samples at 22 ˚C, 20 ˚C and 18 ˚C. Samples incubated at 18 ˚C were analysed using an elution buffer at half the volume of samples at 20 ˚C. Pools of mosquitoes were fed spiked blood containing USUV at a titre of 4 × 10^7^ PFU/ml and were tested by real-time RT-PCR. Saliva samples at 0 dpi were taken immediately after blood feeding and residual virus present in mouthparts likely contaminated saliva as it was expectorated giving rise to false-positive results (A). Here, the proportion of saliva positive samples is forced to one. Body samples at 0 dpi contained virus in the blood meal and so the proportion of body positive samples was 1 at 0 dpi (B).

## Data Availability

Data supporting the main conclusions of this study are included in the manuscript.

## Abbreviations

Dpi: Days post infection
EPI: Extrinsic incubation period
RT-PCR: Real-time reverse transcription polymerase chain reaction
USUV: Usutu virus
WNV: West Nile virus

## Declarations

**Ethical approval and consent to participate.**

Not applicable.
