# Supplementary material for: Impact of temperature on vector competence of Culex pipiens molestus: implications for Usutu virus transmission in temperate regions
Source: Parasit Vectors. 2025 Jul 29;18:310. doi: 10.1186/s13071-025-06948-z (PMC12309054; doi:10.1186/s13071-025-06948-z)
Supplement: Supplementary file 1 — Additional file 1. Figure A1: Proportion of body and saliva positive samples at 22 ˚C and 20 ˚C. Saliva samples at 0 dpi were positive, likely from virus contamination in the mouthparts, and so here are forced to one (A). Body samples at 0 dpi were positive, likely from virus in the bloodmeal (B). Pools of mosquitoes were fed spiked blood containing USUV at a titre of 4 × 10^7^ PFU/ml and were tested by real-time RT-PCR. [file 13071_2025_6948_MOESM1_ESM.docx]

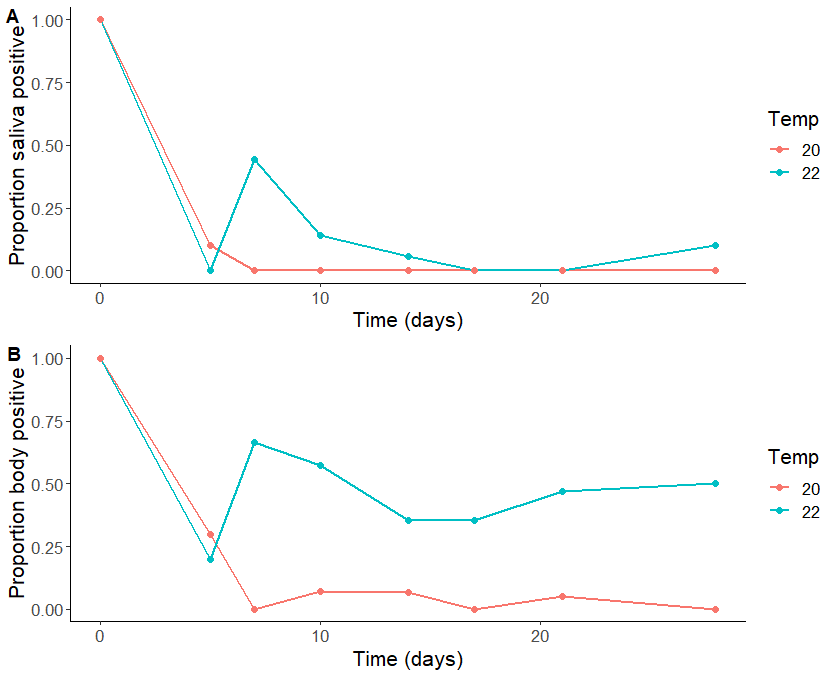


Figure A1: Proportion of body and saliva positive samples at 22 ˚ C and 20 ˚ C. Saliva samples at 0 dpi were positive, likely from virus contamination in the mouthparts, and so here are forced to one (A). Body samples at 0 dpi were positive, likely from virus in the bloodmeal (B). Pools of mosquitoes were fed spiked blood containing USUV at a titre of 4 x 10^7^ PFU/ml and were tested by real-time RT-PCR. Mosquito samples were collected at 0 dpi (n = 5 [22 ˚ C]; n = 9 [20 ˚ C]), 5 dpi (n = 5 [22 ˚ C]; n = 10 [20 ˚ C]) , 7 dpi (n = 9 [22 ˚ C]; n = 15 [20 ˚ C]), 10 dpi (n = 7 [22 ˚ C]; n = 14 [ 20 ˚ C]), 14 dpi (n = 17 [22 ˚ C]; n = 15 [20 ˚ C]), 17 dpi (n = 17 [22 ˚ C]; n = 17 [20 ˚ C]), 21 dpi (n = 17 [22 ˚ C]; n = 20 [20 ˚ C]) and 28 dpi (n = 10 [22 ˚ C]; n = 19 [20 ˚ C]).
